# Photoelectronic Properties of End-bonded InAsSb Nanowire Array Detector under Weak Light

**DOI:** 10.1186/s11671-021-03476-4

**Published:** 2021-01-21

**Authors:** Xiaomei Yao, Xutao Zhang, Tingting Kang, Zhiyong Song, Qiang Sun, Dongdong Wei, Jin Zou, Pingping Chen

**Affiliations:** 1 State Key Laboratory of Infrared Physics, Shanghai Institute of Technical Physics Chinese Academy of Sciences, Shanghai, 200083 China; 2 University of Chinese Academy of Sciences, 19 Yuquan Road, Beijing, 100049 China; 3 Materials Engineering, The University of Queensland, Brisbane, QLD 4072 Australia; 4 Centre for Microscopy and Microanalysis, The University of Queensland, Brisbane, QLD 4072 Australia; 5 School of Physical Science and Technology Northwestern, Polytechnical University, Xi’an, 710129 China

**Keywords:** InAsSb, NW, Photodetector, Weak light, Room temperature

## Abstract

A simple fabrication of end-bonded contacts InAsSb NW (nanowire) array detector to weak light is demonstrated in this study. The detector is fabricated using InAsSb NW array grown by molecular beam epitaxy on GaAs substrate. The metal-induced gap states are induced by the end-bonded contact which suppresses the dark current at various temperatures. The existence of the interface dipole due to the interfacial gap states enhances the light excitation around the local field and thus upgrades the photoresponsivity and photodetectivity to the weak light. The light intensity of the infrared light source in this report is 14 nW/cm^2^ which is about 3 to 4 orders of magnitude less than the laser source. The responsivity of the detector has reached 28.57 A/W at room temperature with the light (945 nm) radiation, while the detectivity is 4.81 × 10^11^ cm·Hz^1/2^ W^−1^. Anomalous temperature-dependent performance emerges at the variable temperature experiments, and we discussed the detailed mechanism behind the nonlinear relationship between the photoresponse of the device and temperatures. Besides, the optoelectronic characteristics of the detector clarified that the light-trapping effect and photogating effect of the NWs can enhance the photoresponse to the weak light across ultraviolet to near-infrared. These results highlight the feasibility of the InAsSb NW array detector to the infrared weak light without a cooling system.

## Introduction

As an important branch of narrow band-gap III–V semiconductors, InAsSb has the benefits inherited from InAs such as small electron effective mass and high electron mobility [1, 2]. When the Sb element is introduced into InAs, the cutoff wavelength response of ternary InAsSb could be extended to long-wavelength infrared range owing to the bandgap bowing effect [3]. Consequently, InAsSb is considered as an ideal candidate in the area of infrared detection [4–6]. In the field of optoelectronics, one-dimensional (1D) nanostructures [7] have tremendous unique properties including large surface area with numerous trap states, long path length for photon absorption and mechanically flexible structure due to their huge aspect ratios [8]. Additionally, during their development, 1D nanostructures may easily release the lattice mismatch to substrates and in turn achieve high crystal quality [9]. Herein, the applications for optoelectronics such as photodetectors [10], solar cells [11] based on 1D nanostructures are attracting tremendous research interests. Among them, the tailored device structures [12] were brought up to achieve optimized light absorption and broadband light harvesting, making 1D nanostructures suitable for varied application scenario and achieving the compatible components to silicon-based integrated circuits. Recently, photodetectors based on individual InAs NWs have demonstrated their potentials in infrared detection [13]. With the addition of Sb, ternary InAsSb can be accessible across the broad-spectrum range for room temperature detection [14]. With the passivation of Al_2_O_3_, detectors based on InAsSb NWs have achieved an uncool detection to the mid-wavelength infrared spectrum [15]. However, conventional light sources widely applied in these studies are high-intensity lasers and most of these devices cannot operate at room temperature [16]. Besides, the conventional device structure based on NWs is not friendly to the mass application in compatible integrated circuits. There are three main types of traditional detectors based on InAsSb NWs, including individual NW device [17], quantum wells embedded in InAs NWs [3] and vertical individual NW device [14]. All of them are required costly nanofabrication process, such as electron beam lithography (EBL) and reactive ion etching (RIE). Herein, the innovation in the structure of device is urgent for the application of NWs.

The interface always plays a vital role in the optical and electrical performance of the device despite the limited size, making contact engineering in NW-based devices another essential factor [18]. For example, solar cell with excellent omnidirectional photodetection properties to weak light was achieved in the hybrid structure utilizing the interface between graphene quantum dots and polystyrene sulfonate [19]. In this study, we modulate the optoelectrical performance of the device using the band structure at the interface between electrode and semiconductor. The charge redistribution happens at the metal–semiconductor interface, and the charge transfer occurs between metal and the tails of metal wave functions into the semiconductor. The redistribution is referred to as MIGS, which could induce gap states and interface dipole at the interfacial states [20]. However, the simulation results from the MIGS model still have deviations between experiments, which is considered resulting from interface defects, fabricated induced defects and Fermi-level pinning [21]. Especially for InAsSb NWs with rich surface states, the Fermi-level pinning is inevitable so that the gap states induced would filter the charge transfer. In this way, the dark current of the device could be suppressed in an acceptable range. Furthermore, the interfacial dipole could enhance the light excitation in the local field which is essential for the weak light detection. Based on the discussion by Chu. et.al, the end-bonded junctions are more likely to achieve the state overlap between metal and semiconductor than the planar-bonded junctions [18]. Yet, the end-bonded device via individual NW is faced with the obstacles in fabrication. Here, we come up with a solution by using NW arrays to achieve the end-bonded contact between NWs and metal. Compared to the conventional photodetectors, the sandwich-structured NW array devices have the advantages of easy manufacturing and high environmental adaptability [22, 23]. The filler (AZ5214), which is spin-coated and baked around the NWs during the fabricated process, makes the device more stable and anti-oxidant to the environment. When the light is introduced in the NW array, it reflects and refracts in different directions multiple times, boosting the absorption of light inside [24, 25]. The prolonged light path in the NW array is referred to as the light-trapping effect [26, 27], which is widely used in NW-based array devices. Both the band structure and the device structure give the device potential for the weak light detection at room temperature.

In this study, we fabricated the NW array device based on the InAsSb NWs grown by MBE (molecular beam epitaxy). The gap states and interface dipole induced by the metal–semiconductor contact could suppress the dark current and boost the light detection separately [28]. The light-trapping effect of the sandwich structure of the NW array contributes to the weak light detection [29]. Suppressed dark current at room temperature greatly minimizes the detection noise and enables the detection at room temperature [30]. The curing device structure makes the device able to work at ambient environment. Also, the photoresponse of the device fluctuates with temperature because the complicated interface states are anticipated. With a constant temperature, the value of photocurrent increases linearly with the incident light intensity, which demonstrated potential for the optical power meter.

## Methods/Experimental

InAsSb NWs were grown on GaAs {111}B substrates using the MBE system (Riber 32 R&D) with an in situ Au evaporate system. The epi-ready substrate was pretreated to remove the contamination. Then, a GaAs buffer layer was deposited at 540 °C for 15 min and the Au nanoparticles were formed via the evaporating and annealing process. InAs stems were grown for 20 min with the temperature maintained constantly at 380 °C, and then Sb source was introduced to the growth chamber for 60 min. During the NW growth, the In BEP (beam equivalent pressure) was kept as 2.7 × 10^–7^ mbar, the As BEP was 2.2 × 10^–6^ mbar, and the Sb BEP was 7 × 10^–7^ mbar, leading to a V/III flux ratio of ~ 11 and the Sb/As ratio of ~ 0.3.

For the device fabrication, AZ5214 (photoresist) was employed as the propping agent to maintain the orientations of NWs. Then the NW array with the substrate was spin-coated with AZ5214 at 3000 rpm for 30 s and baked for 2 min at 120 °C. The gel AZ5214 is transparent that minimizes the light loss during the measurement. To expose the tips of the NWs, the surface of the array was polished by a precision shear gauge (Logitech). The InAsSb layers formed on the substrate surface during the NW growth via VS (vapor–solid) mechanism can act as the drain electrode. According to the Hall measurement of InAsSb epi-layers (shown in Additional file 1: Figure S1), the room temperature carrier concentration is about 2 × 10^17^ cm^−3^, while the mobility is about 1.6 × 10^4^ cm^2^/(V·s) at room temperature. After that, Au film with a nominal thickness of 8 nm was deposited on the selected areas, one of them is on the top of the array, and the other is on the epi-layer. The small thickness of deposited Au assures the electrode photopermeability and acceptable light loss during the measurements.

The morphological, chemical and structural characteristics of obtained InAsSb NWs were investigated using SEM (FE-SEM, JEOL 7800F) and TEM [TEM, Philips Tecnai F20, equipped with energy disperse spectroscopy (EDS) for compositional analysis]. Individual NWs for TEM analysis were prepared by ultra-sonicating the NW samples in ethanol and dispersing them onto the Cu grids supported by carbon films.

The photoconductivity measurements were taken in a helium closed-cycle cryostat equipped with LEDs as light sources. The temperature in this system can be modulated between 2 K and room temperature continuously, while the light intensity of LEDs can be tuned easily by the input current. LEDs with variety wavelengths including 260 nm, 620 nm and 945 nm were employed in this study. The light intensity of LED is related to both temperature and the input current. The intensity increases linearly with current and decreases with the temperature. The values of light intensity at room temperature in this measurement are 4000 nW/cm^2^ for 260 nm, 558 nW/cm^2^ for 620 nm and 14 nW/cm^2^ for 945 nm. The related light intensity information can be found in Ref. [30]. A constant voltage V_DS_ = 100 mV was applied between the source and drain. Photoconductivity response can be obtained through tuning the ON/OFF of the LEDs.

## Results and Discussion

**Fig. 1:**
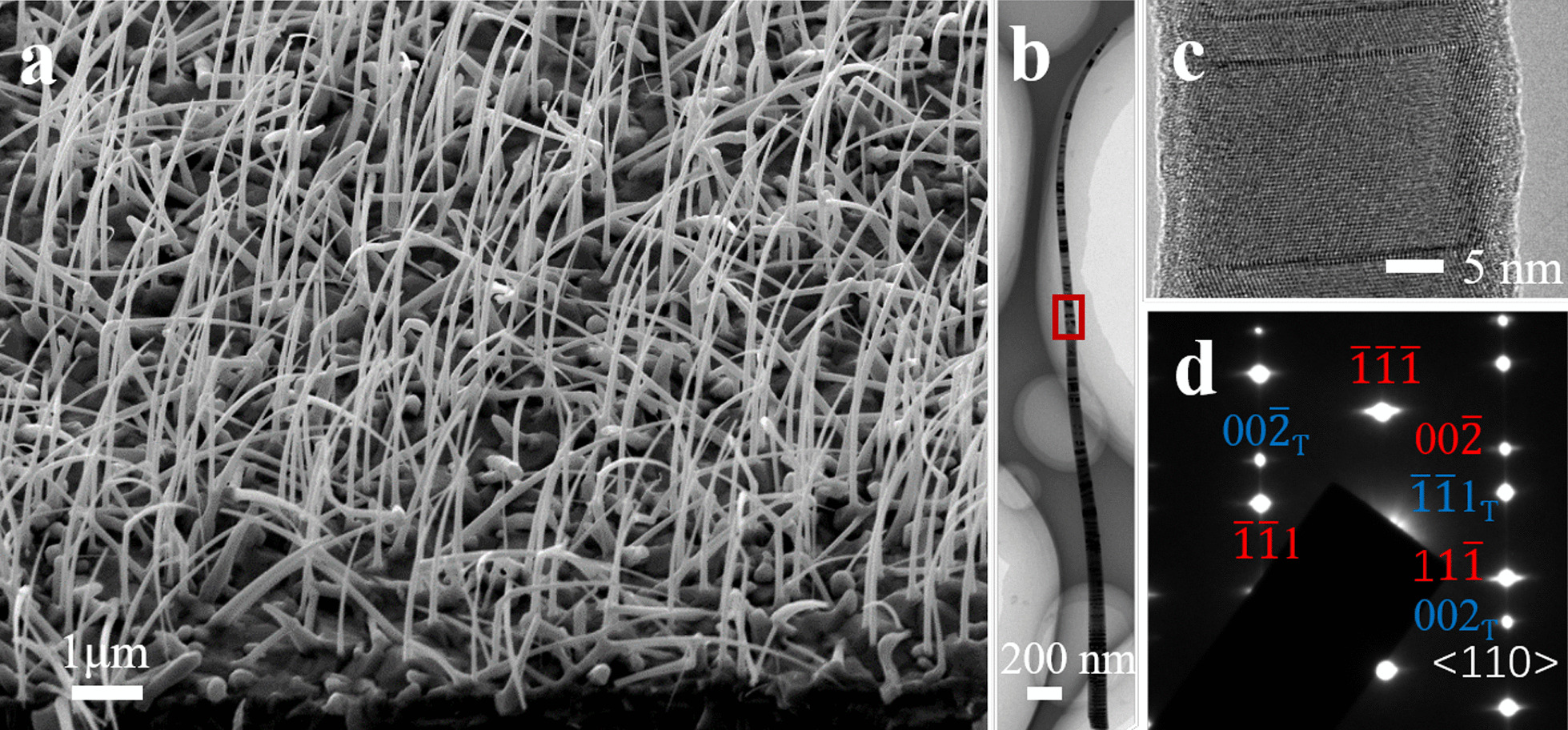
Advanced electron microscopy investigations on InAsSb NWs. **a** A tilted-view SEM image of the NWs. **b** Bright-field (BF)TEM image of an individual NW. **c** High-resolution TEM (HRTEM) image taken from the middle of the NW from the marked region in **b**. **d** Corresponding SAED (selected area electron diffraction) pattern taken from **c**

Figure 1 shows electron microscopy investigations of the InAsSb NWs. Figure 1a is a tilted-view SEM image, showing the diameters of the NWs range from 100 to 200 nm and the length ranges from 6 to 8 μm. Figure 1b shows a bright-field (BF) TEM image of a typical individual NW, indicating a classic tapering structure. Along its axial direction, the composition of the NW shows a moderate gradual change and the average Sb concentration is high up to 30% based on our quantitative EDS analysis (details available in Additional file 1: Figure S2). Figure 1c shows the HRTEM image of the middle part of the NW, confirming the existence of the twin planes. The selected area electron diffraction (SAED) pattern shown in Fig. 1d also verifies the twinning structure, and two sets of ZB (zinc blende)-structured diffractions can be distinguished. The Sb element can be used as a surfactant and depresses the WZ (wurtzite) phase of InAs NWs [31], favoring the structure phase change from WZ to ZB. In our case, the V/III ratio is ~ 11, leading to a V-rich environment, which favors the nucleation of ZB structure [32], but leaving a few twin planes. The investigation about the twinning structure in InAsSb NWs claimed that the displacement at the boundary would cause an uneven local Sb distribution [12], favoring electron scattering or carrier trapping [33].

**Fig. 2:**
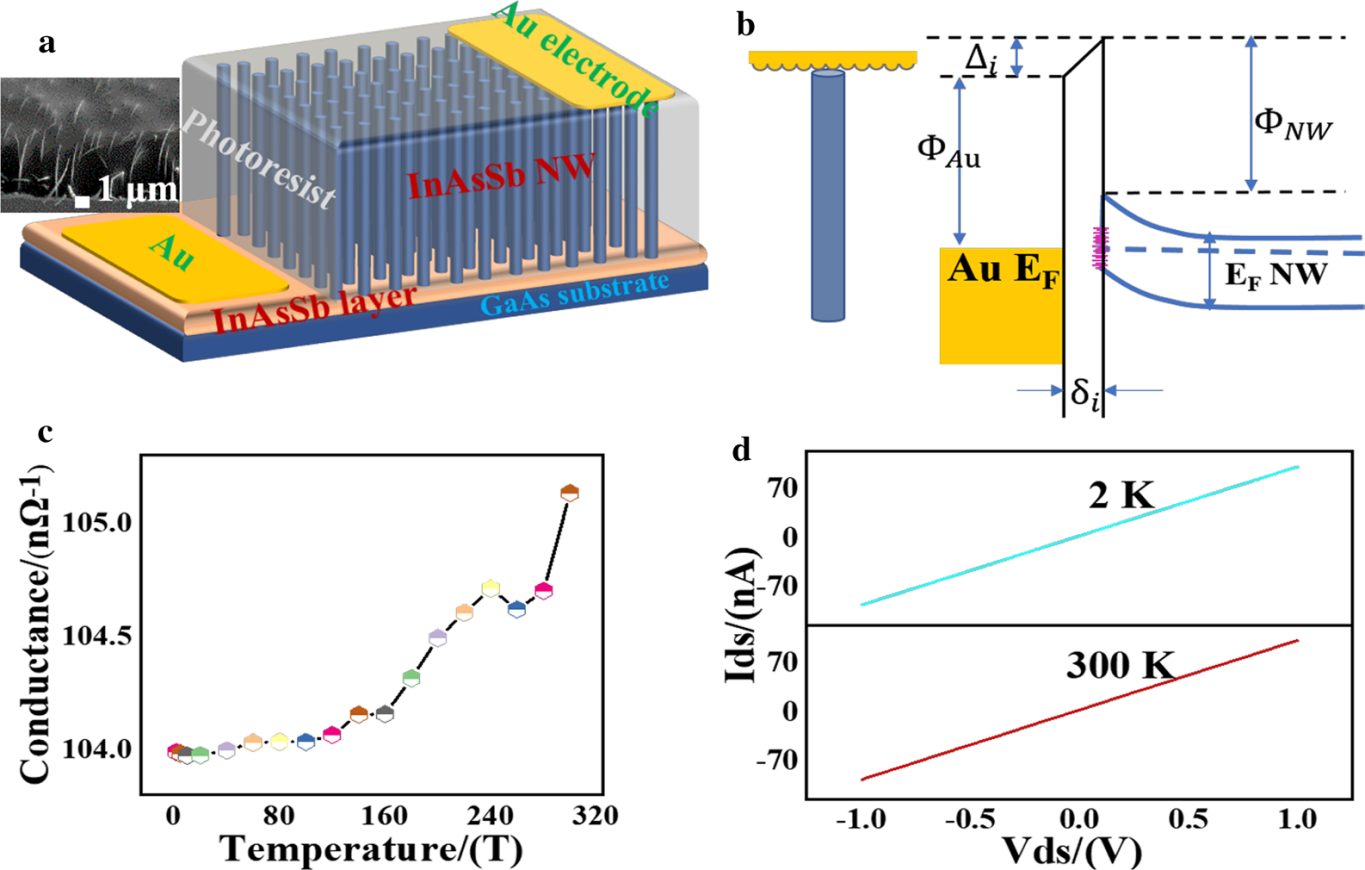
Structure and electrical properties of the InAsSb NW array device. **a** Sketch map of the device, with the SEM image shown in the inset. **b** The energy diagram of the Au-InAsSb interface states. **c **The temperature-dependent conductance of the device. **d** I–V curves at 2 K and 300 K without light, respectively

The device structure is illustrated in Fig. 2a, in which the Au film covers the top and the bottom of the array. The SEM image of the device is shown in Additional file 1: Figure S3, where the remaining length is about 3 µm and almost all the NWs are integrated. The photoresist is used to keep the NWs orientational and integrate the NWs into a curing device; in this way, the device is more anti-oxidation and suitable for the application. The tapering structure is used in amorphous silicon NWs array device, demonstrating an enhancement absorption and is insensitive to the incident angle [34]. Figure 2b is a sketch map of the Au-InAsSb interface determined by the MIGS model. Figure 2c, d confirms a nearly constant conductance independent with temperature, and the value of the conductance is about 1 × 10^–7^ Ω^−1^. The I–V curves at 2 K and 300 K without incident light are shown in Fig. 2d. The individual nanowire has a much bigger value of the conductance shown in Additional file 1: Figure S4. The array device is equivalent to a parallel circuit which joined by thousands of individual NWs so that the theoretical conductance should have been a much more considerable value. Additionally, we have two basic knowledge about the conductance-related issues: (1) the conductance of individual NW shows a strong dependence on the temperature; (2) the array devices made of InAs NWs in our study also have constant conductance. Hence, we conclude that the contact between the metal and the semiconductor in this device has a considerate resistance dominating the overall output characteristics.

When Au is joined in the InAsSb NWs via an end-bonded contact, the charge transfer takes place at the interface via the tails of the metal electron wavefunctions which is called the continuum of MIGS [18]. The charge redistribution at the interface occurred once the contact forms would cause interface dipoles to develop as well [35]. According to the MIGS model, the interface barrier height is determined by ${\Phi }_{\mathrm{Au}}$ (the work function of the metal), ${\Phi }_{\mathrm{NW}}$ (the electron affinity of the InAsSb NW) and ${\Delta }_{i}$ (the voltage drop due to an interface dipole which occurs upon the formation of the interface). The ${\delta }_{i}$ is the distance of the gap states induced by the metal. The electronic state is displayed in Fig. 2b. The interface dipole could create an extra barrier for electrons [36], yet the effect is constrained in a region of the ${\delta }_{i}$. Above all, the intrinsic properties of the device are modulated by the large parasitic contact resistance [37]. In our device, the large contact resistance decreases the dark current effectively, while the value is independent of the temperature. In this way, the carrier concentration can be restricted in a favorable range for light detection. Yet the mechanism of contact resistance due to the interface dipole keeping constant with variety temperatures remains more detailed research.

**Fig. 3:**
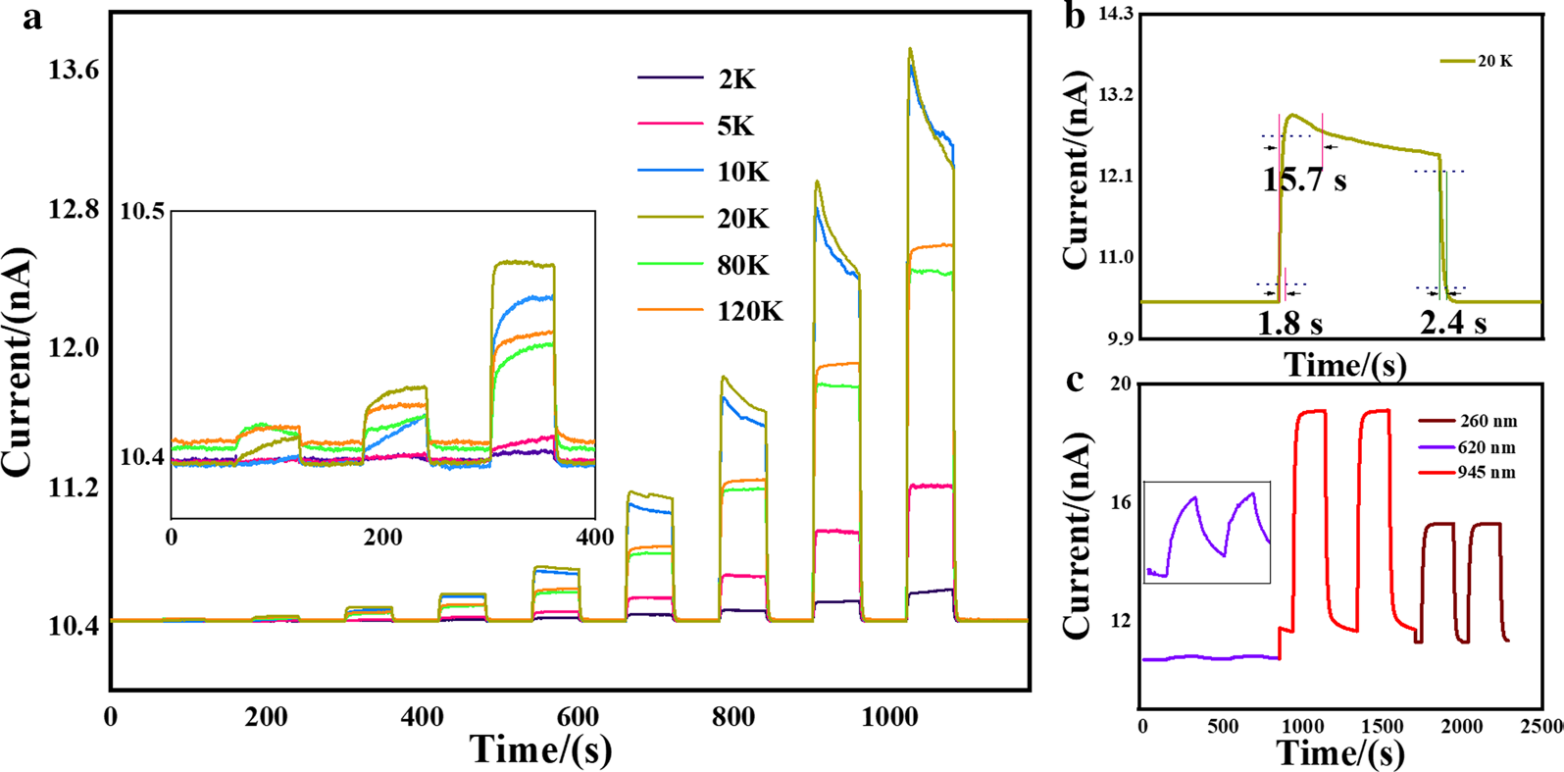
Time dependence of the source-drain conductance under different temperatures. **a** The photoresponse of the device at different temperatures to 620 nm LED with the different input current. **b** The response time of the device at 20 K, while the input current of LED is 2000 μA. **c** The wavelength dependence photoelectrical performance of the device at room temperature with the illumination of 260 nm, 620 nm and 945 nm. The inset shows an enlarged version of the photoresponse to 260 nm LED

In Fig. 3a, we display the currents of the device from 2 to 120 K with and without the light illumination, and the rest is displayed in Additional file 1: Figure S5. The states of LED are tuned with time, in which the states of “ON” and “OFF” would keep for 60 s, respectively. The specific current values of the LED shown at the “ON” states are 10, 20, 50, 100, 200, 500, 1000, 2000 and 3000 uA, respectively. The measurement is taken at different temperatures from 2 to 300 K. The inset in Fig. 3a shows conditions with the three weakest light (about 4–10 nW/cm^2^) illumination, indicating a similar tendency with Fig. 3a. However, obvious different optical behaviors can be stated with the weakest light, especially for the slower response speed and a slightly persistent photoconductance. Figure 3b shows the response time of the device at 20 K, while the current of the LED is 2000 μA. Worth noting that, Fig. 3c is obtained in the ambient environment at room temperature. More importantly, the light source we used here is all LEDs, and the values of light intensity are 4000 nW/cm^2^ (260 nm), 558 nW/cm^2^ (620 nm) and 14 nW/cm^2^ (945 nm), respectively. Apart from the photoresponsivity, from the response speeds with different light wavelengths, we can conclude that the InAsSb NWs have a better response to the infrared light.

Figure 3a shows that our device has a fast and obvious positive response upon the change of LED states under different temperatures, and the photoconductance increases with the LED current. Without light illumination, the conductance of the device is about 1.04 × 10^–7^ Ω^−1^, which agrees with the output test shown in Fig. 2c. At a fixed temperature, the value of Δ*G* (defined as the conductance minus the dark conductance) increases almost linearly with the LED current representing the light intensity. When the light source was blocked, the current of the device immediately recovers to the originate state. The maximum value of Δ*G* in this plot is 3.2 × 10^–8^ Ω^−1^ at 10 K. It should be noted that two kinds of photoresponse can be distinguished in this plot:

1. for most temperatures, the currents have a rapid increase once the LED is turned on, and the currents are stable as long as the LED is on;
2. at 10 K and 20 K, the current has a rapid increase with the light illumination as well. Yet the current has a slight decrease with the LED on, leaving a tail in the plot, which is not found in other temperatures.

To clarify the intrinsic mechanism in the two different kinds of photoresponse, the information of response speed at 20 K when the current of LED is 2000 μA is extracted as the evidence and the specific values are shown in Fig. 3b. The response time [38, 39] (*τ*_ris_ representing the time gap from the 90% current peak to the 10% current peak) is determined as 1.8 s, while the recovery time (*τ*_rec_ defined oppositely) is 2.4 s, which are nearly a constant in the entire temperature range. For 10 K and 20 K, the delay time of the “tail” structure is about 15.7 s, which is surprisingly absent when the illumination of LED current is smaller than 500 μA. Combined with the weak light condition in the inset of Fig. 3a, three kinds of photoresponse at 10 K and 20 K can be demonstrated. With the weakest light (10–50 μA), the current slowly increases with time. When the LED current is increased to 100–500 μA, the response becomes fast. Once the current is over 1000 μA, the tail forms. In other words, only sufficient light intensity may trigger the transient “tail” structure. Similar “tail” structures have been widely reported in InAs NWs [40, 41]. The light sources in these reports are all lasers with a high light intensity, which is consistent with our result that the “tail” structure only appears in the high-intensity light zones. They claimed that the “tail” comes from the lagged effect that corresponds to the trapping and de-trapping of carriers on the surface states [42]. For InAsSb NWs in our case, the surface states are more inevitable due to the severe surfactant effect incorporated with Sb [43]. Hence, we anticipate that the “tail” structure originates from the defect states of the twinning structure only trap electrons at certain temperatures with strong enough light assistance.

For a given photodetector, the photoresponsivity can be expressed by [44]

1 $$\mathrm{R}=\frac{{I}_{\mathrm{p}}}{PA}$$

where ${I}_{\mathrm{p}}$ is the photocurrent of the device, $P$ is the light power on the device, and $A$ represents the effective area of the device. For our device, the effective area of the device is 1 mm^2^ which is determined by the mask used during the electrodes evaporation, and the light-receiving area of the photometer is 0.9 cm^2^. Under this circumstance, the photoresponsivity of the device can be determined to be 4.25 mA/W (260 nm), 1.27 A/W (620 nm) and 28.57 A/W (945 nm), respectively, which further confirmed the potential of InAsSb NW sandwich-structured device in infrared detection.

The photodetectivity of a device can be presented as [14]

2 $${D}^{*}=R{A}^\frac{1}{2}/{(2e{I}_{\mathrm{dark}})}^\frac{1}{2}$$

where *R* is the photoresponsivity of the device and *e* is the electronic charge. *I*_dark_ represents the dark current of the device, and the value is 10.8 nA. With the suppressed dark current in InAsSb NW sandwich structure, the values of ${D}^{*}$ of the photodetector reach 7.28 × 10^7^ (260 nm), 2 × 10^10^ (620 nm) and 4.81 × 10^11^ cm·Hz^1/2^ W^−1^ (945 nm), respectively. Notably, the duty ratio of NWs in this array structure is smaller than 50%, which makes the actual *R* and ${D}^{*}$ bigger than the result we calculated. The high *R* and ${D}^{*}$ are not only attributed to the light-trapping effect of the array device but also originated from the interface structure [2]. Compared to the nanowire-based photodetectors summarized in Ref. [45], the operation temperature of 300 K for our device has superior application potential in real-world scenery [6]. Additionally, at room temperature range, photoresponsivity of our easy-fabricated InAsSb NW array device (28.57 A/W at 945 nm) could exceed most complicated NW-based devices (WSe_2_/Bi_2_Te_3_: 20 A/W at 980 nm [46], PtSe_2_/perovskite: 0.12 A/W at 800 nm [47]). Even though the interface dipole is experimental unreachable, the output characteristics in Fig. 2 could provide solid evidence for its existence in our device. As suggested in the previous discussion, the interfacial layer of the device could function as the optical dipole lattice with the light illumination, which could contribute to a larger field enhancement factor. This effect is referred to as interface dipole enhancement effect (IDEE) in previous studies [48]. The IDEE works for a larger wavelength range than the surface plasmon enhancement effect which only exists within the resonance wavelength range. The enhancement effect around the interfacial states and the light-trapping effect of the array device work together for the weak light detection in our device.

**Fig. 4:**
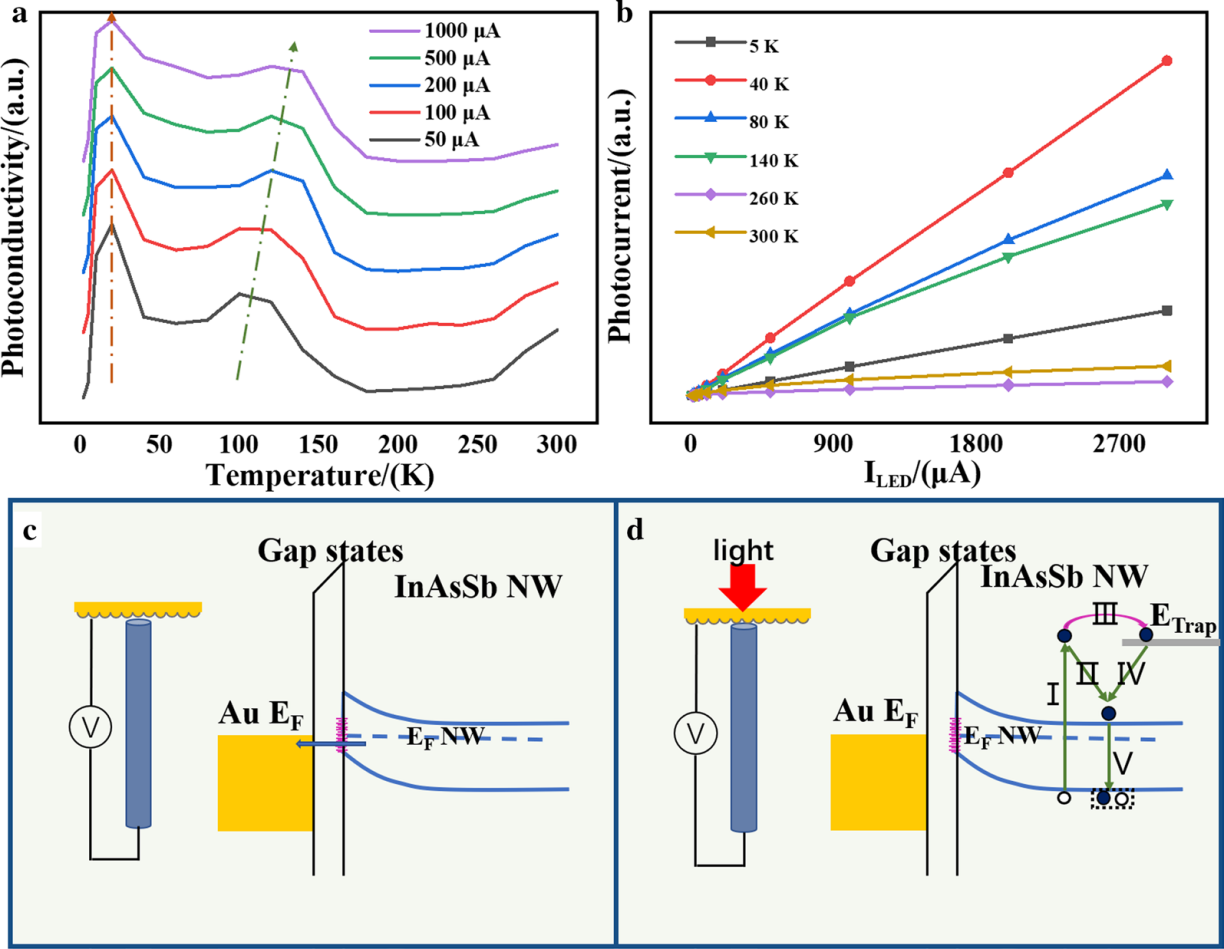
**a** The temperature dependence of Δ*G* measured with different light intensities. **b** The power dependence of Δ*G* measured at different temperatures. **c** The band structure of the device with bias voltage. **d** The band structure of the device with the light illumination

Figure 4 shows the relationship between the photoresponse of the InAsSb NW device as a function of temperatures (Fig. 4a) and the light intensity (Fig. 4b). The value of ${I}_{p}$ is the extreme value the photocurrent can get with the light on. The photoresponse is normalized by the exact light intensity to shield its influence on the tendency. At first, we can conclude a similar tendency with different light intensity illumination. In all plots, the absolute photoconductance increases from 2 to 20 K and then decreases till 80 K, leaving the first peak around 20 K and the second peak around 100–120 K. The temperature range of this peak is in agreement with the specific temperature range where the transient photocurrent “tail” exists. The other peak is around 100–120 K, and its specific location shifts to a higher temperature zone with increasing light intensity.

The photocurrent could be expressed by [28]

3 $${I}_{p}=qg{V}_{\mathrm{NW}}[\tau {\mu }_{d}/l]$$

where $q$ is the elemental charge, $g$ is the photocarrier generation rate, ${V}_{\mathrm{NW}}$ is the NW volume, $\tau$ is the minority carrier lifetime, and ${\mu }_{d}$ is the drift mobility and $l$ is the NW length. This equation clarifies the minority carrier lifetime and drift mobility are two key parameters for the photocurrent [43]. The optoelectrical process of the InAsSb array device is shown in Fig. 4c, d. Before the light imported, the electrons transfer between Au and InAsSb NWs forms the dark current. The gap states due to the interface dipole are short enough for the carrier transfer with enough momentum. In our device, the interface states induced by the native twinning structure and the fabricated-induced defects can both act as trapping states. Upon the light on, the excess electrons with enough energy and momentum would be trapped on the interface states as shown in processes I and III. The decreased electron concentration makes the mobility in the channel increase and prolongs the lifetime of the photogenerated electrons. On the other hand, the trapped electrons in the interface state scatter the electrons in the channel and make the mobility decrease. The released electrons would get back to the conductance band through process IV and participate in the current. The electrons with lower energy would be motivated onto the conductive band and participate in the current as shown in process II. After a while, the electrons would recombinant with the holes left in the valence band as shown in process V. We can conclude two kinds of scattering processes in the device: the trapping electrons scattering centers and electron–electron scattering in the channel [49]. More trapping electrons on the interface states would decrease the carrier mobility and the carrier concentration in the channel. Subsequently, the electron–electron scattering would be weakened and act on the increase of mobility in turn. In conclusion, these two scattering processes would cooperate on the current and get an extreme around 10–20 K. The notable feature of this peak is the “tail” structure, the stable peak site and the persistent photoconductance with the ultraweak light illumination. With the ultraweak light illumination, the induced quantity of the photons is not sufficient to reach the saturated photocurrent at once. Therefore, the device shows a persistent photocurrent until saturation. When the light intensity is increased, the photoexcited carriers boost the current and reach the extreme value within a short response time. However, it is more complicated with higher light intensity. The excess carriers over the saturated states become trapped in the interface states. When the trapped electrons released to the conductance band, the concentration increases again. The increasing electron–electron scattering makes the current decrease, which is referred to as a lagged effect, and creates the “tail” structure.

For the second peak around 100–120 K, a similar peak shift has been reported in the Bi_2_Te_3_ film [50]. Our analysis indicates the existence of recombination centers in this temperature range. The intrinsic mechanism is similar to Bi_2_Te_3_, both related to the balance of photocurrent (${I}_{\mathrm{p}}$) and dark current (${I}_{\mathrm{d}}$). In our case, ${I}_{\mathrm{d}}$ is nearly constant in the whole measurement temperature range. ${I}_{\mathrm{p}}$ is determined by the minority carrier lifetime and drift mobility. Noted that these two parameters of InAsSb NWs have opposite-dependent relationships with temperature. For the minority carrier lifetime, the thermal excited dark carriers increase with the temperature as well as the recombination rate of the photogenerated carriers [51]. Thereby, the minority carrier lifetime is inversely proportional to temperature. The drift mobility is proportional to temperature as the high temperature arises the thermal excitation effect in the NWs. The peak emerges when ${I}_{\mathrm{p}}$ and ${I}_{\mathrm{d}}$ achieve balance at a certain temperature, which is at about 100–120 K. With a higher light intensity, a bigger amount of photogenerated carriers would need more thermal excited carriers at a higher temperature to require balance. Hence, the second peak shifts to a higher temperature when the light intensity increases. Figure 4b shows the light intensity dependence photoconductivity of the InAsSb NW device, where the Δ*G* values are not normalized. As can be seen, the light intensity of the LED strictly increases linearly with the input current (refer to Additional file 1: Figure S6). Hence, this result represents the relationship between the photoresponse and the light intensity, demonstrating the potential of the InAsSb NW array device in optical power meter.

## Conclusions

In summary, the sandwich-structured photodetector based on InAsSb NW array has achieved a splendid optical performance due to the MIGS induced by the end-bonded contact. Interface dipole and gap states suppress the dark current and enhance detection ability of the device. The native defects and the fabricated-induced defects in the device act as the interface states to modulate the optical properties. Even with the ultraweak light (4–20 nW/cm^2^) illumination, the device shows obvious photoresponse at room temperature. The response to LEDs with different wavelengths indicated that the InAsSb NW array device has the strongest response to the infrared light (945 nm). The photoresponsivity and photodetectivity are 40 A/W and 7 × 10^11^ cm Hz^1/2^ W^−1^, respectively. These results confirmed that the sandwich structure in this study favors the repeatability and reliability of the NW devices, which paves a way for the fabrication of NW-based devices. Most importantly, the device may work in an ambient environment at room temperature, which is a great breakthrough for infrared detection.

## Supplementary Information

**Additional file 1:** Temperature dependent carrier concentration and mobility of InAsSb epilayer. HRTEM image of individual InAsSb nanowire. XRD result of InAsSb NW array. SEM image of the device. Output character of individual NW device. Photo-response of the device in other temperatures. Light intensity information of the LED.

## Data Availability

All data are fully available without restriction. The datasets used and/or analyzed during the current study are available from the corresponding author on reasonable request.

## Abbreviations

MBE: Molecular beam epitaxy
1D: One-dimensional
NW: Nanowire
BEP: Beam equivalent pressure
VS: Vapor–solid
VLS: Vapor–liquid–solid
EBL: Electron beam lithography
RIE: Reactive ion etching
WZ: Wurtzite
ZB: Zinc blende
BFTEM: Bright-field scanning electron microscope
HRTEM: High-resolution transmission electron microscope
SAED: Selected area electron diffraction
EDS: Energy disperse spectroscopy
MIGS: Metal-induced gap state
IDEE: Interface dipole enhancement effect
LED: Light emitting diode
