# Supplementary material for: Photoelectronic Properties of End-bonded InAsSb Nanowire Array Detector under Weak Light
Source: Nanoscale Res Lett. 2021 Jan 21;16:13. doi: 10.1186/s11671-021-03476-4 (PMC7818373; doi:10.1186/s11671-021-03476-4)
Supplement: Supplementary file 1 — Additional file 1: Temperature dependent carrier concentration and mobility of InAsSb epilayer. HRTEM image of individual InAsSb nanowire. XRD result of InAsSb NW array. SEM image of the device. Output character of individual NW device. Photo-response of the device in other temperatures. Light intensity information of the LED. [file 11671_2021_3476_MOESM1_ESM.docx]

**Photoelectronic Properties of End-bonded InAsSb Nanowire Array Detector under Weak Light**

**Xiaomei Yao^1, 2, 3^, Xutao Zhang^1, 5^, Tingting Kang^1^, Zhiyong Song^1^, Qiang Sun^3^, Dongdong Wei^1^, Jin Zou^3,4^ and Pingping Chen^1, 2*^.**


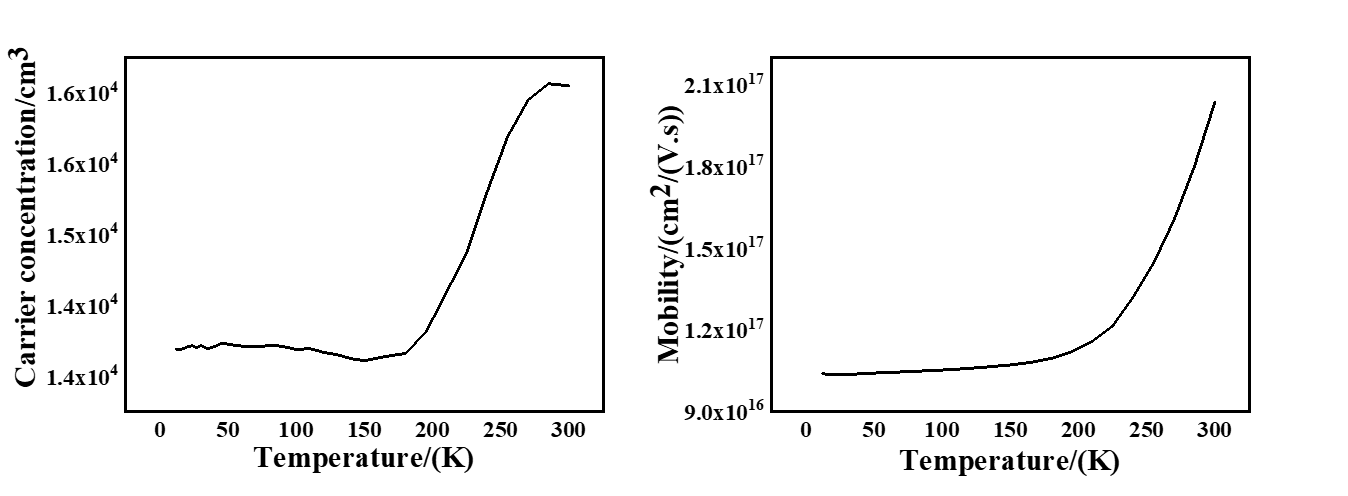
 **Figure S1:** (a) The temperature dependent carrier concentration of the InAsSb epilayer. (b) The temperature dependent mobility of the InAsSb epilayer.


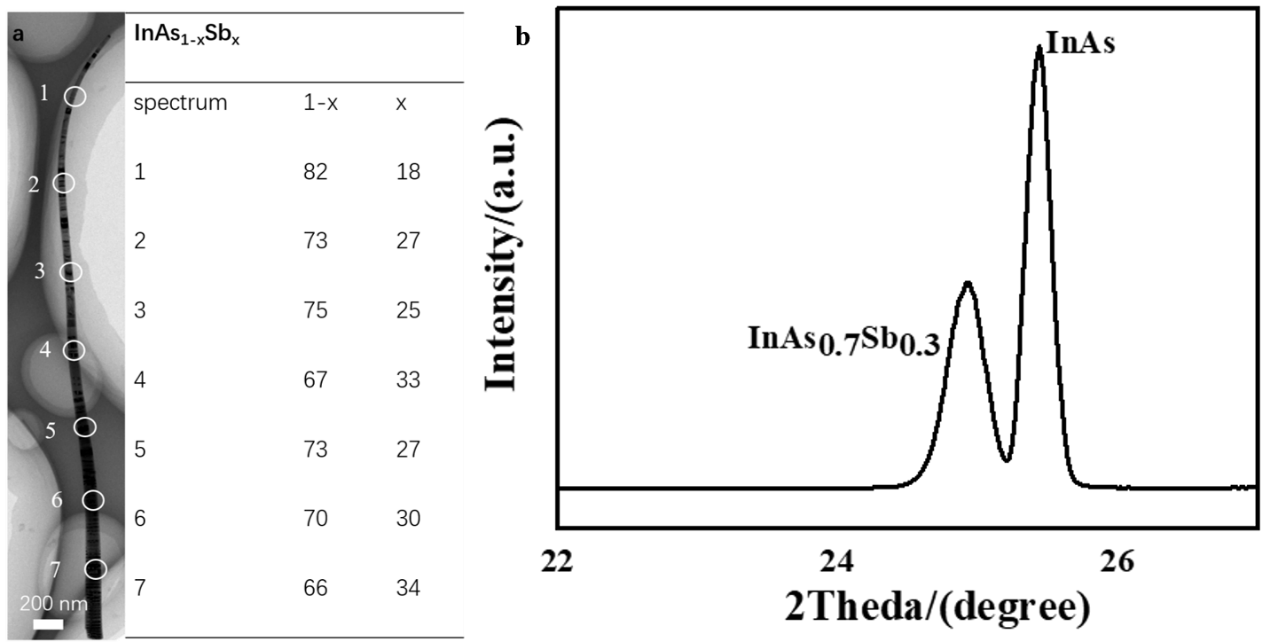


**Figure S2:** (a) The HRTEM image of the individual InAsSb nanowire and the value of the Sb content of the spot shown in (a). (b) XRD result of the InAsSb NW array with substrate.


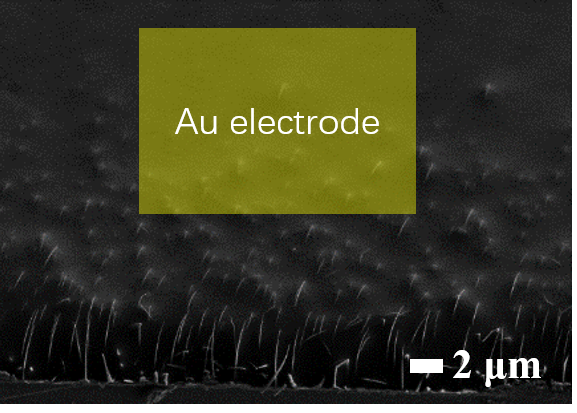


**Figure S3:** The SEM image of the device.


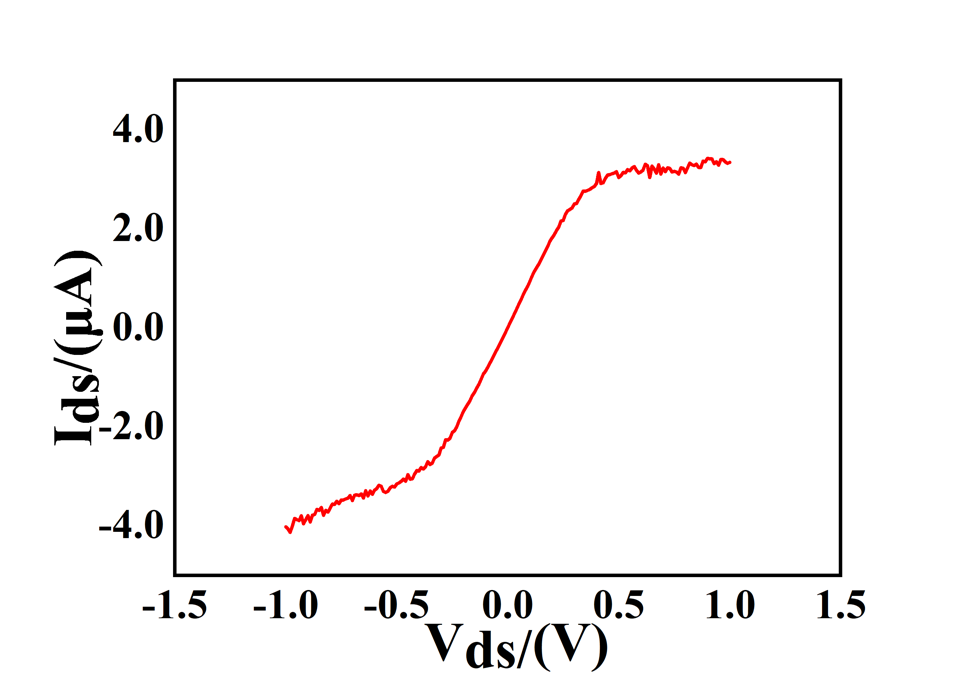


**Figure S4:** The output character of the individual NW device.


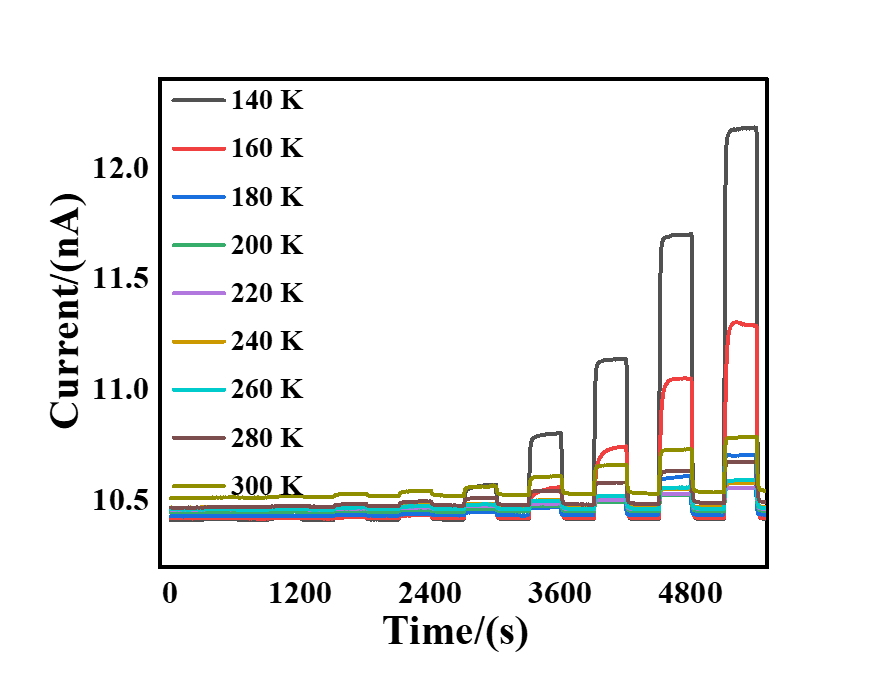


**Figure S5:** The photo response of the device in other temperatures. The inset shows the response time.


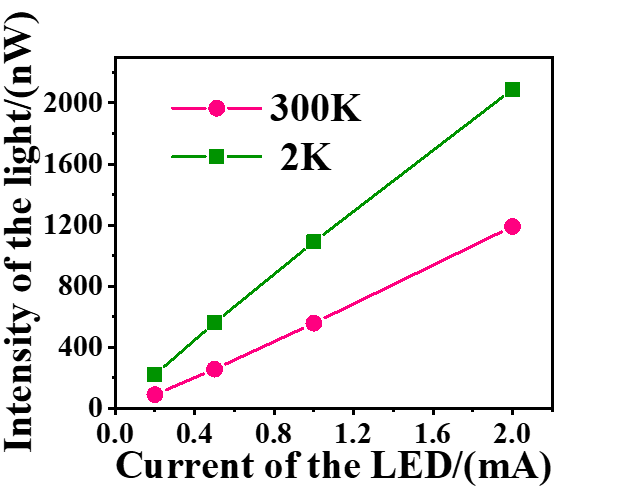


**Figure S6:** The relationship between the light intensity of the LED (620 nm) and the input current.
